# BRCA Testing in Prostate Cancer: A Histopathologist’s Perspective

**DOI:** 10.3390/diagnostics16152358

**Published:** 2026-07-27

**Authors:** Francesca Sanguedolce, Roberta Mazzucchelli, Vincenza Conteduca, Brigida Anna Maiorano

**Affiliations:** 1 Unit of Pathology, Department of Clinical and Experimental Medicine, University of Foggia, Policlinico Riuniti, 71122 Foggia, Italy; 2 Section of Pathological Anatomy, Department of Biomedical Sciences and Public Health, United Hospitals, Università Politecnica delle Marche, 60131 Ancona, Italy; r.mazzucchelli@staff.univpm.it; 3 Unit of Medical Oncology and CREATE Center for Research and Innovation Medicine, Department of Medical and Surgical Sciences, University of Foggia, Policlinico Riuniti, 71122 Foggia, Italy; vincenza.conteduca@unifg.it; 4 Department of Medical Oncology, Comprehensive Cancer Center, IRCCS San Raffaele, 20132 Milan, Italy; brigidamaiorano@gmail.com

**Keywords:** prostate cancer, BRCA testing, histopathology, homologous recombination repair, intraductal carcinoma

## Abstract

BRCA1/2 alterations have emerged as clinically relevant biomarkers in prostate cancer because of their prognostic significance and predictive value for response to poly(ADP-ribose) polymerase inhibitors. As indications for molecular testing expand, histopathologists play a pivotal role in ensuring the quality and reliability of somatic BRCA testing. This narrative review provides a histopathologist-oriented overview of the principal pre-analytical, technical, and morphological issues influencing BRCA testing in PC, with emphasis on tissue selection, specimen processing, nucleic acid preservation, and genotype–phenotype correlations. The success of somatic BRCA testing depends on several pre-analytical variables, including fixation, storage time, tumor cellularity (generally ≥10–20% neoplastic cells for reliable NGS analysis), specimen type, and DNA quality. Bone metastasis specimens require special attention because decalcification procedures may compromise nucleic acid integrity and molecular testing performance. From a morphological perspective, BRCA-associated PCs, especially those harboring BRCA2 alterations, are characterized by adverse clinicopathological features, including a higher grade and stage at diagnosis. Although intraductal carcinoma of the prostate and cribriform architecture are associated with genomic instability and poor prognosis, their value as surrogate markers of BRCA1/2 alterations remains uncertain. Histopathologists play a central role in optimizing BRCA testing through appropriate specimen selection, tissue handling, and multidisciplinary collaboration, thereby supporting precision medicine in prostate cancer.

## 1. Introduction

Prostate cancer (PC) is the second most frequently diagnosed malignancy and one of the leading causes of cancer-related mortality among men worldwide [1]. Although the majority of cases follow a relatively indolent clinical course, a clinically significant subset exhibits aggressive biological behavior characterized by rapid progression, metastatic dissemination, and poor clinical outcomes [2]. The recognition of the molecular heterogeneity underlying PC has profoundly transformed the diagnostic and therapeutic landscape of the disease, shifting the focus from purely clinicopathological risk stratification toward a more integrated molecular approach capable of identifying biologically distinct subgroups with specific prognostic and therapeutic implications [3].

Among the molecular pathways involved in prostate carcinogenesis, alterations affecting the homologous recombination repair (HRR) system have emerged as highly relevant. The HRR pathway plays a crucial role in maintaining genomic stability through the accurate repair of double-strand DNA breaks. Defects in HRR-related genes result in the accumulation of genomic damage, increased mutational burden, and progressive genomic instability, ultimately contributing to tumor initiation and progression [4,5]. Among these genes, BRCA2 represents the most frequently altered HRR gene in advanced PC, followed by BRCA1 and other DNA damage repair (DDR) genes. Both germline and somatic alterations have been associated with aggressive clinicopathological features and unfavorable clinical outcomes, highlighting their relevance as biomarkers of disease behavior [6,7,8,9,10].

The clinical importance of BRCA testing has increased substantially following the introduction of poly(ADP-ribose) polymerase (PARP) inhibitors and the demonstration of their efficacy in patients harboring HRR gene alterations. In the pivotal phase III PROfound trial, olaparib significantly improved radiographic progression-free survival compared with enzalutamide or abiraterone in patients with metastatic castration-resistant prostate cancer and qualifying HRR alterations, thereby establishing BRCA1/2 alterations as clinically actionable predictive biomarkers [11,12]. Consequently, BRCA assessment is no longer limited to hereditary cancer risk evaluation but has become an essential component of therapeutic decision-making in advanced PC [2,13,14,15]. Furthermore, the progressive expansion of testing indications toward earlier disease stages and broader patient populations is expected to further increase the number of specimens requiring molecular characterization.

Within this evolving scenario, the histopathologist plays a pivotal role. Unlike germline testing, which is typically performed on peripheral blood samples, somatic BRCA testing relies predominantly on formalin-fixed paraffin-embedded (FFPE) tissue specimens obtained during routine diagnostic practice. The success of molecular analyses is therefore highly dependent on multiple pre-analytical, analytical, and post-analytical variables that fall, at least in part, under the responsibility of the pathology laboratory [16,17]. Specimen selection, fixation procedures, tissue processing, tumor cellularity assessment, nucleic acid preservation, and management of challenging samples such as decalcified bone metastases may all significantly influence the quality of molecular results and ultimately determine whether a patient can access targeted therapies [17]. The widespread implementation of next-generation sequencing (NGS) has further emphasized the importance of appropriate tissue selection and pre-analytical quality. Since somatic BRCA testing relies predominantly on FFPE specimens, several technical factors may directly influence the success of molecular analysis, highlighting the need for close integration between surgical pathology and molecular diagnostics. These aspects are discussed in detail in the following sections [16,18,19].

In addition to technical issues, considerable attention has recently been devoted to the relationship between histological phenotype and underlying molecular alterations. While several morphological features have been associated with aggressive disease and genomic instability, their ability to predict BRCA1/2 alterations remains an area of active investigation [9,20,21,22].

Despite the growing literature addressing the clinical and therapeutic implications of BRCA1/2 alterations in PC, relatively limited attention has been paid to the practical challenges encountered by histopathologists in the implementation of BRCA testing and in the interpretation of morphology–genotype correlations. Given the expanding role of molecular diagnostics in routine pathology practice, a pathology-oriented perspective is increasingly needed to optimize specimen management, improve testing success rates, and facilitate appropriate patient selection for molecular analysis.

The aim of this narrative review is to provide a comprehensive histopathologist-focused overview of BRCA1/2 testing in prostate cancer. Although other homologous recombination repair (HRR) genes and related molecular biomarkers are discussed where relevant, they are considered only insofar as they provide the biological and clinical context necessary for interpreting BRCA testing in routine pathology practice. Emphasis is placed on pre-analytical and technical factors influencing test performance, the advantages and limitations of different tissue sources, and the current evidence regarding genotype–phenotype correlations, including the controversial relationship between BRCA1/2 alterations and intraductal or cribriform morphology.

## 2. BRCA Somatic Testing in Prostate Cancer: Practical Challenges and Pitfalls

In current clinical practice, somatic BRCA testing is primarily performed on FFPE tissue specimens obtained during routine diagnostic work-up [17,23]. Compared with fresh or frozen tissue, FFPE samples undergo a series of processing procedures aimed at preventing autolysis, in order to preserve tissue architecture (through formalin fixation), while providing adequate mechanical support for the preparation of extremely thin sections suitable for microscopic examination (through paraffin embedding), after histological staining. When properly standardized in their sequence and timing, these procedures ensure optimal preservation of both tissue morphology and biomolecular integrity, allowing reliable histopathological assessment as well as ancillary investigations, including immunohistochemistry (IHC), in situ hybridization (ISH), and molecular analyses. Conversely, suboptimal tissue processing may induce significant molecular alterations that can compromise the reliability of downstream genetic testing [16].

A recent review addressing the challenges associated with germline and somatic BRCA testing in PC reported failure rates for somatic mutation detection in FFPE samples ranging from 6.7% to 38% across the studies analyzed, compared with a failure rate of approximately 3% for blood-based testing in the only study evaluating this approach [16]. Several factors contribute to the variability in DNA yield and quality obtained from FFPE tissues, including specimen type (e.g., core needle biopsy (CNB) versus surgical specimen), tumor volume, and tumor cellularity, usually expressed as the percentage of neoplastic cells within the analyzed sample [17]. Although minimum tumor cellularity requirements vary according to the analytical platform and laboratory validation, a tumor content of at least 10–20% neoplastic cells is generally considered adequate for reliable NGS-based testing. When tumor cellularity is limited, macrodissection may increase the proportion of neoplastic cells and improve analytical performance. In addition, intratumoral heterogeneity should be considered during specimen selection, as a single tissue sample may not fully represent the genomic landscape of advanced prostate cancer.

Another critical determinant of successful somatic BRCA testing is specimen storage time. In a recent multicenter study including 954 FFPE samples from 11 Italian institutions, including both primary and metastatic biopsies as well as surgical specimens, sequencing success rate (SSR) was inversely correlated with specimen age. Successfully sequenced samples showed a significantly shorter median storage time compared with failed samples (657 vs. 1626.5 days; *p* < 0.001). Moreover, SSR was significantly associated with higher DNA concentration, a higher DNA fragmentation index (a surrogate marker of DNA integrity), and the use of surgical specimens rather than biopsies. Based on the latter finding, the authors suggested preferential use of surgical specimens for somatic testing whenever available, as they are more likely to provide superior DNA integrity and overall quality [24].

Similarly, Tommasi et al. demonstrated a progressive reduction in DNA extraction yield across three groups of histological samples stratified according to storage time (<3 years, 3–5 years, and >5 years; *p* = 0.0006). In the same study, DNA quality, measured using the DNA Integrity Number (DIN), was significantly associated with storage duration, with low-quality samples exhibiting a longer mean storage time than medium-quality samples (4.0 vs. 2.9 years, respectively; *p* = 0.006) [25]. Comparable results were reported by Hussain et al. in an analysis of more than 4000 cases of metastatic castration-resistant prostate cancer (mCRPC) enrolled in the phase III PROfound trial. Although molecular testing remained feasible in 47.3% of samples stored for more than 10 years, NGS success rates progressively declined with increasing specimen age. Consistent with the observations of Vescovo et al., NGS success was also significantly associated with specimen size, ranging from 52.4% in CNB to 69.8% and 74.0% in transurethral resection of the prostate (TURP) and radical prostatectomy (RRP) specimens, respectively [26].

Collectively, these observations support the notion that prolonged storage adversely affects nucleic acid preservation in FFPE tissues. The progressive degradation and fragmentation of DNA over time reduce the probability of obtaining sufficient amounts of high-quality genetic material, thereby negatively impacting the performance and success rates of downstream molecular assays.

The quality of extracted DNA is profoundly influenced by pre-analytical tissue handling, particularly fixation procedures. Neutral buffered 10% formalin remains the standard fixative for routine histopathological specimens because of its ability to preserve both tissue morphology and antigenicity. Notably, fixation practices require strict standardization, which is not always achievable in routine clinical settings. Tissue degradation begins early during the ‘warm ischemia’ phase, defined as the interval between surgical interruption of blood supply and specimen removal, and continues during ‘cold ischemia’, when the specimen remains at room temperature or under cooling conditions before complete fixation [27,28].

To minimize the detrimental effects of these delays on nucleic acid integrity, fixation should be initiated as soon as possible after tissue removal and ideally within 20 min [29]. Appropriate fixation time is essential to preserve DNA and RNA integrity, in order to ensure reliable molecular testing. In general, fixation times between 8 and 24–48 h are recommended, depending on specimen size [27,28]. Because formalin has a limited tissue penetration capacity, large surgical specimens should be adequately sectioned to increase exposure to the fixative. In addition, replacement of the fixative after several hours may be beneficial, especially in large specimens containing substantial amounts of biological fluids such as blood or inflammatory exudate, which can dilute formalin and reduce its fixation efficiency.

Underfixation, defined as consistently delayed or shorter fixation duration, may result in incomplete tissue preservation and subsequent enzymatic degradation, whereas overfixation may promote excessive cross-link formation and impair nucleic acid extraction [27,28]. Formaldehyde is known to induce multiple forms of DNA damage, including DNA–protein cross-links, DNA–DNA cross-links, histone-associated complexes, and chemical adduct formation. Furthermore, FFPE-derived DNA is specifically susceptible to cytosine and 5-methylcytosine deamination, resulting in uracil and thymine formation, respectively. These alterations may generate sequencing artifacts and false-positive nucleotide variants during NGS analysis [19].

Overall, current evidence supports preferential selection of larger FFPE specimens, which are more likely to contain abundant tumor tissue, as well as samples stored for less than 2–3 years whenever possible. Equally important is the implementation of rigorous pre-analytical standardization protocols, with particular attention to ischemia times and fixation procedures, in order to maximize DNA quality and improve the success rate of NGS-based somatic BRCA testing.

### Practical Workflow for Histopathologists

**Figure 1:**
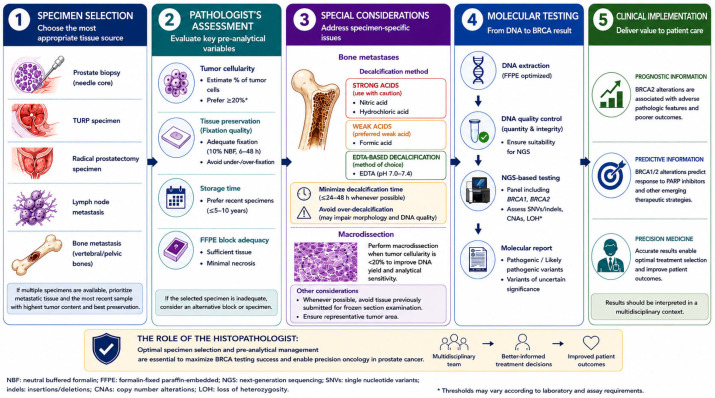
Practical workflow of the histopathologist in somatic BRCA testing of prostate cancer specimens. The infographic summarizes the principal steps involved in routine pathology practice, including specimen selection, pathological assessment of tissue adequacy, pre-analytical considerations, management of challenging specimens, and communication with the molecular laboratory to optimize molecular testing.

A practical workflow for tissue-based somatic BRCA testing is summarized in Figure 1. From the histopathologist’s perspective, the following principles may facilitate specimen selection and optimize molecular testing in routine practice:

• Prioritize metastatic tissue whenever available and technically suitable; otherwise, select the most recent primary specimen with the highest tumor content.
• Choose the most appropriate FFPE block, preferentially selecting recent, well-preserved tissue with limited to no necrosis and/or excessive inflammation, whenever multiple specimens are available.
• Ensure adequate tumor cellularity (generally‚ ≥10–20%, according to laboratory validation) and consider macrodissection when tumor content is limited.
• For bone metastases, preferentially use EDTA-based or other nucleic acid-preserving decalcification protocols and avoid over-decalcification.
• Verify specimen suitability for NGS according to local laboratory quality requirements before molecular testing.
• Interpret molecular findings in conjunction with the clinical and pathological context within a multidisciplinary team.

The detailed rationale supporting each of these recommendations is discussed in the following sections.

## 3. Choosing What to Test: Primary Versus Metastatic Specimens

While some homologous HRR gene alterations may occur during the early stages of prostate carcinogenesis, others may arise and accumulate throughout disease progression. Consequently, careful consideration should be given to the choice of tissue submitted for somatic testing, whether derived from the primary tumor (CNB, TURP, or RRP specimen) or from a metastatic site (lymph node, bone, or other metastatic locations). On the other hand, real-world clinical practice often presents situations in which biopsy of a metastatic lesion may not be feasible because of technical or clinical constraints. Moreover, in the metastatic setting, a single small biopsy may fail to capture the biological heterogeneity of the disease; as a result, it may not be fully representative of the entire tumor mutational landscape [29,30]. For these reasons, both national (Italian) and international guidelines recommend the use of either archival primary tumor tissue or metastatic tissue, depending on specimen availability [5,30,31]. Beyond analytical adequacy, the choice of specimen should also consider its biological representativeness. Although metastatic lesions may better reflect the current genomic landscape of advanced disease, practical constraints and intrapatient tumor heterogeneity often make archival primary tissue the only feasible and clinically acceptable source for molecular testing. Thus, specimen selection should balance analytical feasibility with biological relevance according to the individual clinical scenario.

Data from Hussain et al. showed a numerically lower, although not statistically significant, NGS success rate in primary tumor samples compared with metastatic specimens (56.2% vs. 63.9%, respectively). Among metastatic samples, the lowest and highest success rates were observed in bone (42.6%) and lymph node specimens (74.7%), respectively [26]. These findings are consistent with those reported by Incorvaia et al., who found significantly higher testing failure rates in bone metastases than in lymph node metastases (35.7% vs. 5.3%) [32]. Importantly, in the same study, the proportion of inconclusive versus conclusive testing results did not significantly differ according to specimen type (CNB, RRP, or metastatic tissue; *p* = 0.9) [32]. Similarly, Vescovo et al. reported no significant differences in SSR according to biopsy site, including bone biopsies [24].

Bone metastasis specimens deserve special consideration because their processing is inherently more complex than that of routine histological samples. In order to obtain tissue sections thin enough for microtome cutting, calcium salts must first be removed from the specimen through a decalcification procedure. Traditionally, decalcification is achieved using agents capable of dissolving calcium deposits, including strong acids (e.g., nitric acid or hydrochloric acid), weak acids (e.g., formic, acetic, or picric acid), or chelating agents such as ethylenediaminetetraacetic acid (EDTA) [16]. Although strong acid-based decalcification protocols provide rapid achievement of the decalcification endpoint, they induce substantial nucleic acid degradation. Accordingly, while these specimens usually retain adequate morphological preservation for histopathological evaluation, they are generally unsuitable for downstream molecular applications relying on PCR amplification and DNA sequencing, including NGS-based assays. In contrast, EDTA-based decalcification protocols, which are currently the most widely adopted in diagnostic pathology, require longer processing times, maintenance of a neutral pH throughout the procedure, and the use of relatively thin tissue sections. Nonetheless, they provide significantly better preservation of nucleic acid integrity, making the specimens suitable for both histopathological assessment and downstream molecular analyses, including NGS platforms [27,28]. The main characteristics of the different decalcification methods are summarized in Table 1.

From a practical perspective, bone metastasis specimens also require careful gross examination by the pathologist. In particular, the consistency of the submitted tissue should be assessed, as some specimens may consist entirely, or almost entirely, of soft metastatic tumor tissue despite being submitted as bone resections. In such cases, decalcification may be unnecessary. Such ‘physical’ examination of the specimen, often performed to assess the endpoint of decalcification, can be achieved by bending the specimen or by inserting a pin through the tissue. Alternatively, when a clear distinction can be made between densely mineralized bone and grossly evident tumor tissue, the neoplastic component may be separated from the osseous portion using a scalpel and processed routinely without decalcification, whereas only the remaining mineralized tissue is subjected to decalcification. This approach maximizes nucleic acid preservation and may substantially improve the likelihood of obtaining successful molecular testing results.

Overall, both primary and metastatic specimens may be suitable for somatic BRCA testing when appropriately processed and preserved. When multiple specimens are available, specimen selection should consider not only tissue quality and tumor cellularity, but also the biological representativeness of the disease. For this reason, close collaboration between pathologists, oncologists, and urologists is essential to identify the most informative specimen and maximize the likelihood of successful molecular characterization.

When adequate tissue is unavailable or unsuitable for molecular analysis, circulating tumor DNA (ctDNA)-based liquid biopsy has emerged as a valuable complementary approach for the assessment of BRCA1/2 and other HRR gene alterations in selected patients with advanced prostate cancer, as recognized by current international guidelines. Nevertheless, the present review deliberately focuses on tissue-based BRCA testing from a histopathologist’s perspective. Therefore, analytical aspects and clinical applications of liquid biopsy are beyond the scope of this article.

## 4. Genotype–Phenotype Correlation: Current Evidence

### 4.1. BRCA and Adverse Histological Features

**Figure 2:**
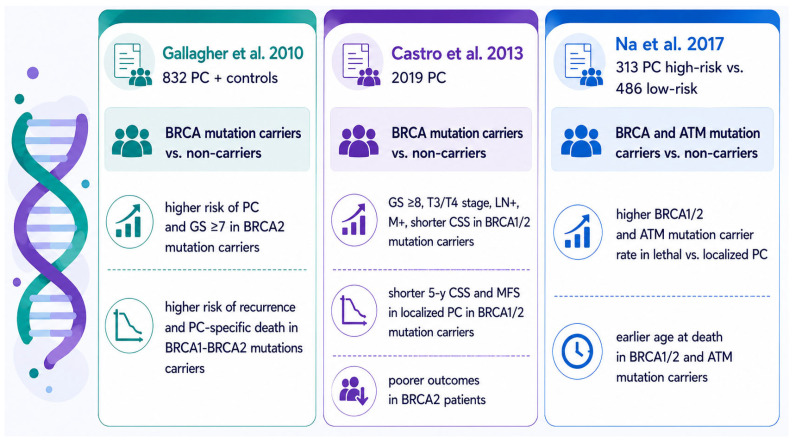
Selected studies reporting an association between BRCA status and unfavorable clinicopathological factors in prostate cancer [6,7,8]. The infographic summarizes the key findings of three landmark studies supporting the association between BRCA1/2 alterations and aggressive disease phenotype and adverse clinical outcomes. CSS: cancer-specific survival; GS: Gleason Score; LN: lymph nodes; MFS: metastasis-free survival; PC: prostate cancer.

A substantial body of clinicopathological evidence supports the association between germline BRCA mutations, with BRCA2 showing the strongest association, and a significantly more aggressive PC phenotype. Carriers of germline BRCA1/2 mutations are more likely to present with advanced-stage disease (T3/T4), lymph node involvement (N1), and distant metastases (M1) at diagnosis. In particular, BRCA2 deficiency has consistently been associated with poorer cancer-specific survival (CSS) and metastasis-free survival (MFS) compared with non-carriers (Figure 2), as demonstrated in a few landmark studies [6,7,8].

From a morphological perspective, BRCA2-associated tumors more frequently exhibit high-grade histological features, including a Gleason Score (GS) ≥7 or ≥8 (corresponding to Grade Group (GG) ≥2–3 and ≥4, respectively). In one study, 85% of BRCA2 mutation carriers harbored poorly differentiated tumors (GS 7–10), compared with 57% of non-carriers [6,7]. Furthermore, alterations affecting DNA damage repair (DDR) pathways, including BRCA2, have been reported to be enriched in tumors displaying a predominant Gleason Pattern (GP) 5, the most aggressive architectural pattern recognized in contemporary grading systems [33].

In contrast, the evidence linking BRCA1 mutations to adverse histopathological features is less consistent. While some studies have suggested a more aggressive clinical course among BRCA1 carriers, others have failed to identify significant differences in GS distribution between BRCA1 carriers and non-carriers [3].

Beyond conventional grading parameters, several studies have explored the association between DDR gene alterations and specific histological variants of PC. Germline DDR mutations have been reported to occur more frequently in tumors exhibiting ductal adenocarcinoma morphology [20,34]. In addition, PCs harboring a ductal component demonstrate a relatively high prevalence of DDR gene alterations, with BRCA2 representing one of the most frequently mutated genes, accounting for approximately 18% of cases in some cohorts [21,35].

BRCA2 alterations have been reported in rare cases of prostate adenocarcinoma with focal pleomorphic giant-cell features, suggesting a possible association with extreme genomic instability; yet, this observation is based on a very limited number of cases [36].

Occasional BRCA2-mutated tumors have been reported to exhibit neuroendocrine/small-cell differentiation, although current evidence remains limited and no statistically significant association has been consistently demonstrated [37]. In the study by Han et al., neuroendocrine/small-cell histology was more frequently observed among BRCA2-mutated patients than among non-carriers (11% vs. 1%), although this difference did not reach statistical significance [37].

Overall, the available evidence suggests that these uncommon morphological variants may occur in BRCA2-altered prostate cancers; nevertheless, the current data are limited and largely derived from small retrospective series. Consequently, these observations should be interpreted with caution until confirmed in larger studies.

### 4.2. Focus on Intraductal and Cribriform Morphology

The current WHO classification defines intraductal carcinoma of the prostate (ID-PC) as a neoplastic epithelial proliferation involving pre-existing, generally expanded duct-acinar structures, displaying architectural and cytological atypia beyond that observed in high-grade prostatic intraepithelial neoplasia (HGPIN) [38]. In the vast majority of cases, ID-PC is associated with high-grade, high-stage PC, although it may occasionally be identified in CNBs in the absence of invasive carcinoma [39]. This finding may be largely attributable to sampling limitations inherent to CNBs, which evaluate only a small fraction of the entire prostate and may thus fail to capture an accompanying invasive carcinoma present elsewhere within the gland.

From a morphological standpoint, ID-PC is a well-recognized mimicker of cribriform carcinoma (CC), the latter representing an architectural growth pattern of GP 4 PC. The differential diagnosis between these two entities is based on the integration of morphological assessment and basal cell immunohistochemistry, the latter relying on the demonstration of the presence of a basal cell layer in ID-PC and its absence in CC-PC [40].

The prognostic significance of ID and CC morphology has been extensively documented. In a Dutch study including 1031 prostate biopsy specimens from patients enrolled in the European Randomized Study of Screening for Prostate Cancer, the presence of ID and/or CC morphology was associated with significantly worse clinical outcomes. In particular, patients with GS 3 + 4 = 7 PC harboring ID/CC morphology exhibited survival rates consistently lower than those observed in patients with GS 6 disease (*p* < 0.001). By contrast, no significant survival difference was observed between patients with GS 3 + 4 = 7 tumors lacking ID/CC morphology and those with GS 6 tumors (*p* = 0.30). Importantly, the association between ID/CC morphology and worse disease-specific survival remained independently significant at multivariable analysis (*p* = 0.002) [41].

**Figure 3:**
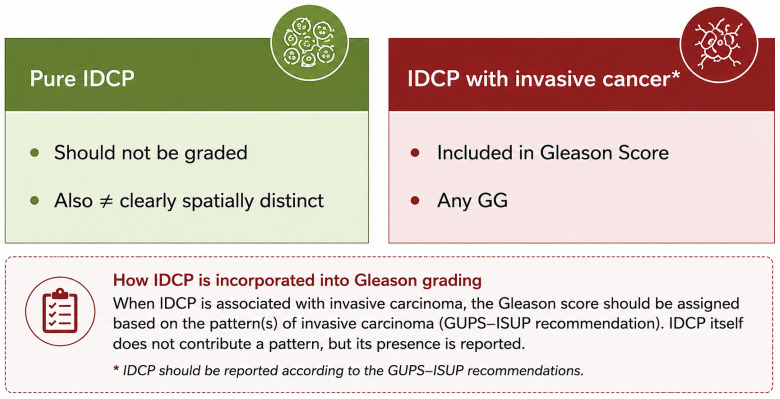
Schematic summary of the GUPS–ISUP recommendations for grading intraductal carcinoma of the prostate (IDCP). The infographic summarizes the current recommendations for incorporating IDCP into the Gleason grading system according to its relationship with invasive prostate cancer.

These findings have subsequently been confirmed by additional studies and comprehensive reviews [41,42], ultimately leading the pathology community to establish consensus recommendations regarding the incorporation of ID-PC into contemporary PC grading systems [43]. The key recommendations emerging from the recent GUPS–ISUP Joint Expert Consultation on ID-PC are summarized in Figure 3. Briefly, isolated ID-PC identified in the absence of invasive PC, or occurring in association with invasive carcinoma but clearly spatially distinct from it, should not be graded. Conversely, when ID-PC coexists with invasive carcinoma, it should be incorporated into the GS regardless of the accompanying GP. Accordingly, ID-PC with cribriform architecture should be assigned to GP 4, whereas ID-PC associated with comedonecrosis should be considered equivalent to GP 5 [44].

Current international recommendations suggest that the presence of ID/CC morphology may represent an additional criterion for BRCA testing in patients with PC. In particular, the NCCN 2026 guidelines for genetic/familial risk assessment in solid tumors, including PC, indicate that genetic testing may be considered in patients with intermediate-risk disease when ID or CC histology is identified [13,14].

**Figure 4:**
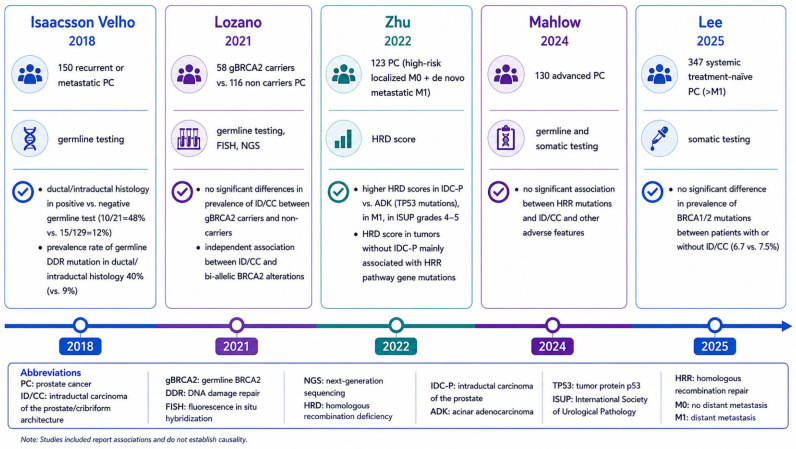
Selected studies focusing on BRCA status in intraductal carcinoma of the prostate [20,21,38,45,46]. The infographic summarizes the principal studies investigating the relationship between BRCA1/2 alterations, intraductal/cribriform morphology, and genomic instability, highlighting the current controversies and inconsistent findings across different patient cohorts.

Despite this recommendation, it is important to distinguish this potential role from the well-established adverse prognostic significance of these morphological patterns, since the association between ID-PC or cribriform architecture and BRCA2 alterations has not been consistently demonstrated across different patient cohorts (Figure 4).

In a seminal study, Isaacson Velho et al. reported a significantly higher prevalence of intraductal and/or ductal morphology among patients with recurrent or metastatic PC carrying germline DDR mutations compared with non-carriers (10/21, 48% vs. 15/129, 12%; *p* < 0.01). Furthermore, among tumors displaying ductal or intraductal morphology, the prevalence of DDR gene alterations was substantially higher than in tumors lacking these features (40% vs. 9%) [20]. Nevertheless, the authors acknowledged that combining ductal and intraductal morphologies into a single category may have represented a significant confounding factor, given that these are biologically and clinically distinct entities.

Subsequently, Lozano et al. analyzed a cohort comprising 58 germline BRCA2 mutation carriers and 116 non-carriers and found no significant differences in the prevalence of ID or CC morphology between the two groups [21]. Nevertheless, both morphologies were independently associated with biallelic BRCA2 alterations, suggesting that complete BRCA2 inactivation may be more closely linked to these histological phenotypes than germline carrier status alone [21].

The molecular basis underlying ID-PC has been further investigated by Zhu et al., who assessed homologous recombination deficiency (HRD) scores in a cohort of 123 patients with high-risk M0/M1 PC. Interestingly, ID-PC was characterized by significantly higher HRD scores than invasive adenocarcinoma. It should be noted that these elevated scores were primarily driven by TP53 alterations, whereas tumors lacking ID-PC more frequently exhibited HRD signatures associated with alterations in canonical HRR genes [39]. These findings highlight the profound genomic instability associated with ID-PC while suggesting that such instability may not necessarily be attributable to BRCA-related mechanisms.

More recent studies evaluating advanced PC cohorts undergoing germline and/or somatic HRR testing have failed to demonstrate a significant association between HRR gene alterations, including BRCA1 and BRCA2, and the presence of ID/CC morphology [45,46].

Several factors may explain the discrepancies observed among these studies. Beyond the intrinsic heterogeneity of the investigated cohorts and differences in clinical characteristics, including the relative representation of metastatic disease, methodological variability likely plays a substantial role. Potential sources of heterogeneity include differences in molecular testing approaches (germline versus somatic testing, HRD score assessment versus NGS-based analyses), source material (blood versus tissue), and the variable diagnostic sensitivity of CNB compared with RRP specimens. In addition, the diagnostic criteria for ID and CC morphology have only recently been standardized, potentially contributing to inconsistencies in case classification across earlier studies [46].

Taken together, the available evidence suggests that although ID/CC patterns are more frequently encountered in tumors characterized by marked genomic instability and are often associated with other adverse clinicopathological features, their value as reliable surrogate markers for predicting BRCA2 alterations or defects in other HRR genes remains incompletely defined. Further studies integrating morphology, molecular profiling, and standardized diagnostic criteria are needed to clarify the biological and clinical significance of these associations [47].

## 5. Conclusions

BRCA testing has become an integral component of the clinical management of prostate cancer, making the histopathologist a key contributor to precision oncology. Appropriate specimen selection, accurate assessment of tumor cellularity, and careful control of pre-analytical variables are essential to maximize the reliability of tissue-based molecular testing.

Bone metastasis specimens require particular attention because decalcification protocols may significantly influence DNA preservation and the success of NGS analysis. From a morphological perspective, BRCA2-altered tumors are frequently associated with adverse clinicopathological features, whereas the value of intraductal carcinoma and cribriform architecture as surrogate markers of BRCA1/2 alterations remains uncertain.

Although additional HRR genes are increasingly incorporated into molecular testing panels, BRCA1/2 currently represent the best-established biomarkers in routine clinical practice. Consequently, optimizing tissue handling and integrating histopathological evaluation with molecular diagnostics remain fundamental to ensuring accurate patient selection and effective implementation of precision medicine in prostate cancer.

## Figures and Tables

**Table 1:** Main characteristics of different decalcification methods.

Decalcification Method	Processing Time	Morphological Preservation	DNA Preservation	Suitability for PCR/NGS
Strong acids (nitric acid, hydrochloric acid)	Short	Good	Poor (marked nucleic acid degradation)	Not recommended
Weak acids (formic acid)	Intermediate	Good	Fair to good	Acceptable for molecular testing
EDTA-based decalcification	Longer	Excellent	Excellent	Method of choice

## Data Availability

No new data were created or analyzed in this study. Data sharing is not applicable to this article.
